# The lipid rafts in cancer stem cell: a target to eradicate cancer

**DOI:** 10.1186/s13287-022-03111-8

**Published:** 2022-08-30

**Authors:** Shuo Zhang, Neng Zhu, Hong Fang Li, Jia Gu, Chan Juan Zhang, Duan Fang Liao, Li Qin

**Affiliations:** 1 Laboratory of Stem Cell Regulation With Chinese Medicine and Its Application, School of Pharmacy, Hunan University of Chinese Medicine, 300 Xueshi Road, Hanpu Science and Education District, 410208 Changsha, Hunan People’s Republic of China; 2 Department of Urology, The First Hospital of Hunan University of Chinese Medicine, Changsha, China; 3 Institutional Key Laboratory of Vascular Biology and Translational Medicine in Hunan Province, Hunan University of Chinese Medicine, Changsha, China; 4 Hunan Province Engineering Research Center of Bioactive Substance Discovery of Traditional Chinese Medicine, Hunan University of Chinese Medicine, Changsha, China

**Keywords:** Lipid raft, Cancer, Cancer stem cell, Self-renewal, Drug resistance, Epithelial-mesenchymal transition, Cancer stem cell niche

## Abstract

Cancer stem cells (CSCs) are a subpopulation of cancer cells with stem cell properties that sustain cancers, which may be responsible for cancer metastasis or recurrence. Lipid rafts are cholesterol- and sphingolipid-enriched microdomains in the plasma membrane that mediate various intracellular signaling. The occurrence and progression of cancer are closely related to lipid rafts. Emerging evidence indicates that lipid raft levels are significantly enriched in CSCs compared to cancer cells and that most CSC markers such as CD24, CD44, and CD133 are located in lipid rafts. Furthermore, lipid rafts play an essential role in CSCs, specifically in CSC self-renewal, epithelial-mesenchymal transition, drug resistance, and CSC niche. Therefore, lipid rafts are critical regulatory platforms for CSCs and promising therapeutic targets for cancer therapy.

## Introduction

According to the Global Burden of Disease (GBD) report in 2017, cancer has been the leading cause of global mortality [1]. Despite advances in cancer prevention, it remains a human nightmare as cures are rare, and most patients have metastasis or recurrence. Cancer stem cells (CSCs) may be to blame because they can resist electromagnetic and chemotherapy through a quiescent state [2]. Meanwhile, as tumor-initiating cells, CSCs supply constant cancer cells for cancers. Therefore, CSCs must be eliminated to eradicate cancer.

Lipid rafts are signaling and transit platforms that are critical in cancer development and progression [3, 4]. Since Simons and Ikonen proposed the lipid raft hypothesis based on the classical membrane fluid mosaic model [5], lipid raft research has gradually deepened. The discussion on lipid rafts at the Keystone Symposium has been agreed upon, “lipid rafts are small (10–200 nm), heterogeneous, highly dynamic, sterol- and sphingolipid-enriched domains that compartmentalize cellular processes. Small rafts can sometimes be stabilized to form larger platforms through protein–protein and protein-lipid interactions” [6]. Lipid rafts, also known as “membrane rafts” or “membrane microdomains,” are divided into caveolae (flask-like invaginations) and planar lipid rafts [7]. Caveolin-1 and flotillins are lipid raft markers [8]. Caveolin-1 is an essential structural protein of caveolae [9], and flotillins are linked to poor cancer prognoses [10].

Cancer cells are previously confirmed to have higher levels of lipid rafts than normal cells [11, 12], while emerging evidence indicates that CSCs contain more lipid rafts than cancer cells. That is, the relative amount of lipid rafts: CSCs > cancer cells > normal cells [13]. Specifically, a study using filipin and conjugated cholera toxin B (CtxB) for plasma membrane cholesterol and GM1 staining showed that the cancer cell lines (PC-3, LNCaP, MCF-7, and MDA-MB-231) have stronger staining than the normal cell lines (PZ-HPV7 and MCF-10A), suggesting higher levels of rafts/ caveolae in the cancer cell lines. Meanwhile, the purified lipid rafts in human prostate cancer cell PC-3 are five times more than in normal cells [11]. Quite similar results have also appeared in melanoma cells, which have 1.5–2 higher lipid rafts levels than non-tumorigenic cells detected by fluorescent probes and flow cytometry [12]. Regarding the lipid rafts of CSCs, the data from CtxB immunostaining and flow cytometry assay have shown that the levels of lipid rafts in CD44^high^ colorectal cancer cells are higher, as the median fluorescence intensity ratio of lipid rafts within CD44^high^ cells is 10%-30% stronger than CD44^low^ cells. Importantly, CtxB^high^ cells showed a greater sphere‐forming capacity [13]. In addition, CD133^+^ pancreatic cancer cells have more caveolin-1and cholesterol than CD133^−^ cells by Western blotting, real-time PCR and Amplex red cholesterol assay, indicating higher levels of lipid rafts in CD133^+^ CSCs [14]. Moreover, lipid rafts are indispensable for CSCs, and our previous study demonstrated that the suppression of lipid raft formation inhibits CD133^+^ CSC stemness [15]. Through transmission electron microscopy observation and sphere formation assay, we found that the reduction in caveolae is accompanied by decreased stemness markers CD133, Bmi-1, SOX-2, and spheroids. Interestingly, CSCs die once the lipid rafts are disrupted. A study revealed that miltefosine and other lipid raft‐disrupting drugs such as MβCD and nystatin downregulate the expression of stem‐related genes and upregulate the expression of differentiation marker genes by RT‐qPCR assay. In parallel, immunofluorescence assays and flow cytometry analysis visually confirmed miltefosine reduces lipid raft levels with a disruption of sphere formation. Similar to in vitro assay, immunostaining assays of xenografted tumors have revealed a decrease in CD44^high^ cells along with an increase in apoptotic cells after miltefosine treatment [13]. Meanwhile, several CSC biomarkers have been found in lipid rafts, which are affected by lipid rafts [16–19]. The above studies suggest that targeting lipid rafts on CSCs is a precise strategy for cancer prevention. The relationship between CSCs and lipid rafts has attracted more and more attention.

## Cancer stem cell: from controversy to confirmation

As defined by the AACR Workshop, CSC is a cell within a tumor that possesses the capacity to self-renew and cause the heterogeneous lineages of cancer cells that comprise the tumor [20]. It is also known as “tumor-initiating cells” or “tumorigenic cells” [21]. The proposition of CSC theory is aggressive and has long been controversial. But now, a wealth of convincing evidence makes the CSC theory even more tenable. In 1994, T Lapidot et al. found a CD34^+^ CD38^−^ cell with the ability to initiate acute myeloid leukemia and self-renewal [22]. Since then, CSCs have been found in brain cancer [23], colorectal cancer [24], osteosarcoma cancer [25], breast cancer [26], colon cancer [27], prostate cancer [28], thyroid cancer [29], ovary cancer [30], pancreatic cancer [31], head and neck squamous cell carcinoma [32], and so on. So far, several CSC markers have been discovered that assist in identifying CSCs. Among them, CD133, CD44, and aldehyde dehydrogenase (ALDH) are markers of most solid tumors [33, 34]. In addition, therapies targeting CSCs are nearing maturity. Anti-CSC drugs are in multiple clinical trials. For example, the chemokine receptor-4 (CXCR4) inhibitor Plerixafor increases chemosensitivity in hematologic malignancies; B-cell lymphoma-2 (BCL2) inhibitors Venetoclax for acute myeloid leukemia can target CSC metabolism [35]. Most importantly, CSCs have several stem cell-like properties (Table 1). First, CSCs have similar markers (such as CD133) to stem cells [36, 37] and a higher telomerase activity just like normal stem cells [38, 39]. In particular, CSCs share self-renewal signaling pathways with normal stem cells [40]. Second, CSCs have self-renewal ability that divides symmetrically into two CSC progenies or asymmetrically into one CSC progeny and one non-CSC progeny [41, 42]. When CSCs stop proliferating, CSCs maintain a quiescent or dormant state and undergo reversible cell cycle arrest to escape therapy [43, 44], resulting in cancer relapse or metastasis. Finally, the CSCs have stem cell-like differentiation potential [45]. CSCs can transform into cancer cells. Meanwhile, they can be transdifferentiated into other cell type [46–48], such as vascular endothelial cells. Cancer cells can also dedifferentiate back into CSCs during appropriate processes such as epithelial-mesenchymal transition (EMT) [49, 50]. That is the plasticity of CSCs, which makes CSCs slyer.

**Table 1:** Similarities and differences between cancer stem cell and normal stem cell

	Cancer stem cell	Normal stem cell	Reference
Similarities	Markers: CD133, SSEA3, SSEA4, TRA-1–60, etc.		[51]
	Biochemical profile: a higher telomerase activity		[38, 40]
	Proliferation: self-renewal (symmetrically and asymmetrically dividing)		[41, 42]
	Differentiation: differentiate into distinct types of cells		[45]
Differences	Markers: CD20, CD96, CD55, and TIM-3 are not markers for normal stem cells but for CSCs		[51]
	Proliferation: CSCs divide more rapidly than normal stem cells (the cell division rate is an order of magnitude larger)		[40]
	Differentiation: CSCs very few undergo terminal differentiation compared to normal stem cells		[52]

**Fig. 1:**
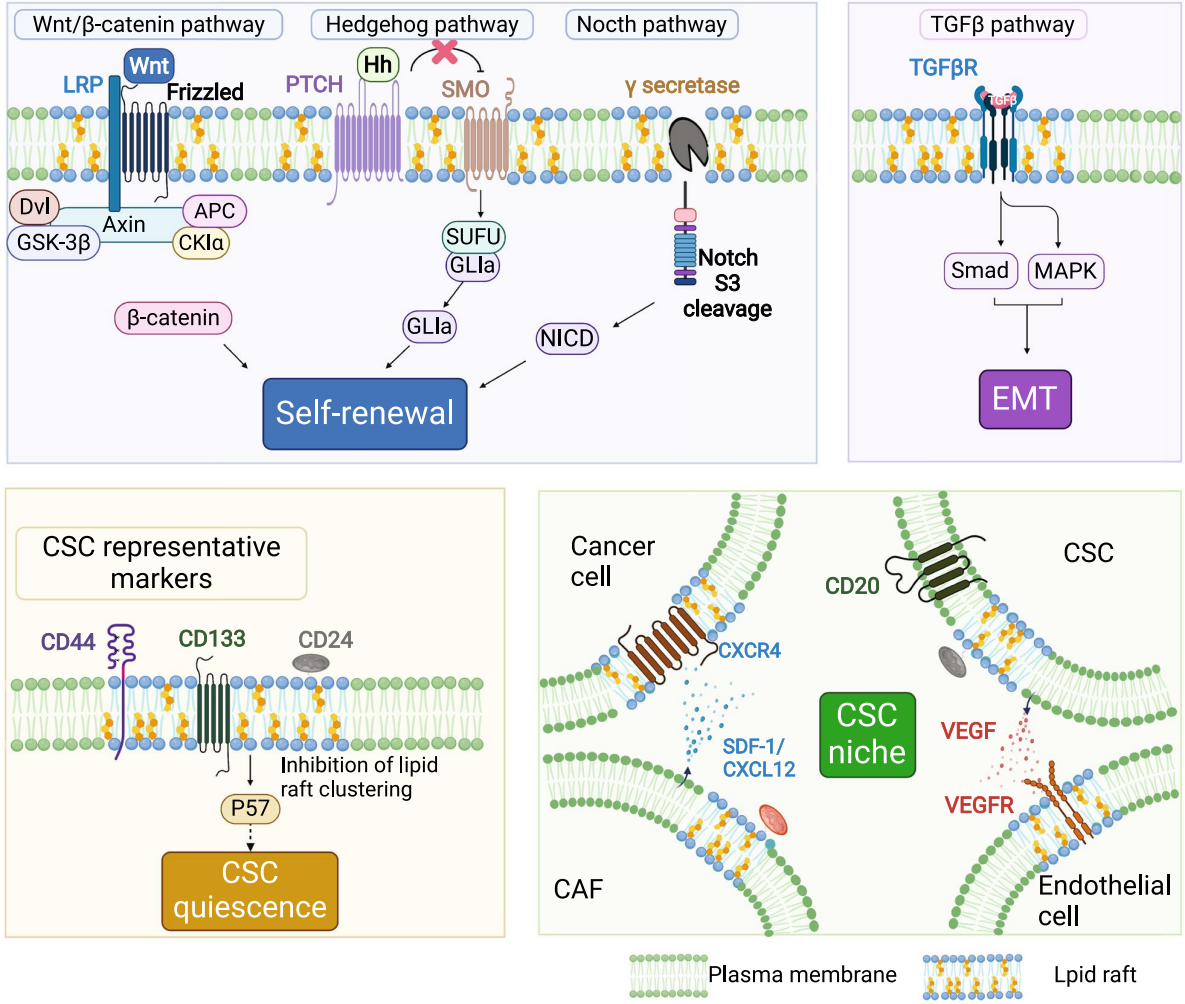
Function of lipid rafts in cancer stem cell. Lipid rafts play a crucial role in cancer stem cells (CSCs). CSC self-renewal: lipid rafts promote CSC self-renewal through the Wnt/β-catenin pathway, Notch pathway, and Hedgehog pathway. Wnt/β-catenin pathway: in lipid rafts, Wnt ligand binds to their receptors Fz8 and LRP, phosphorylate the cytoplasmic domain of LRP, and recruits the scaffolding protein Dvl to disassemble the destruction complex (Axin, APC, GSK-3β, and Ck1α), eventually inhibiting degradation of β-catenin, which activate target gene transcription. Hedgehog pathway: in lipid rafts, the Hh ligand binds to its receptor Ptc, and Ptc releases the repression on Smo, allowing GLI to enter the nucleus and stimulate transcription of downstream target genes. Notch pathway: γ-secretase, the key protease of the Notch receptor, exhibits the highest activity in lipid rafts, which mediates the S3 site cleavage of the Notch receptor, and then releases the Notch intracellular domain (NICD) into the nucleus that promotes transcription of the target genes. EMT: lipid rafts regulate CSC EMT through the TGFβ pathway. The TGF-βRI/ TGF-βRII/ TGF-β signaling complex forms in lipid rafts to activate the downstream signal. CSC quiescence: inhibition of lipid raft aggregation upregulate cyclin-dependent kinase inhibitor p57 (Kip2) expression, which might trigger CSC hibernation. CSC niche: lipid rafts mediate cell communication in the CSC niche. For example, VEGF secreted by CSCs binds to VEGFR2 in lipid rafts of endothelial cells, promoting niche angiogenesis and enhancing self-maintenance; CAFs secrete CXCL12, which is recruited to lipid rafts in cancer cells, where it interacts with CXCR4, regulating CSC plasticity

Although CSCs are not well-studied, they may explain why treatment is ineffective. In brief, CSCs maintain cancer growth through self-renewal capacity and avoids therapy in a quiescent state. The CSC niche (microenvironment around stem cells) provides physical shelter for CSCs and allows non-CSCs to dedifferentiate into CSCs through EMT, resulting in relapse. Therefore, this review evaluates the role of lipid rafts in CSC self-renewal, drug resistance, EMT, and stem cell niche (Fig. 1). An in-depth study of the interrelationships and functions between lipid rafts and CSCs will contribute to designing intervention strategies for cancer eradication.

## CSC markers and lipid rafts

CSC markers are specialized proteins on the CSC surface or secreted by CSCs that help identify or isolate CSCs [51] and predict poor clinical outcomes [53]. In addition, CSC markers are also necessary for CSC functions. Therefore, targeting cell surface markers to destroy CSCs is available [34]. Encouragingly, agents targeting CSC-related markers are already in clinical trials [35], and some have been approved for clinical use. Studies have shown that many CSC markers are located in or associated with lipid rafts, such as CD24, CD44, CXCR4, and CD133 [54, 55]. The relation between lipid rafts and these CSC markers is evaluated as follows.

The glycosylphosphatidylinositol (GPI)–anchored glycoprotein CD24, a typical raft protein, is highly expressed in CSCs and is considered a CSC marker that maintains cancer stemness [56–58]. CD24 can recruit integrin into lipid rafts that facilitate cancer development. Further studies have found that CD24 enhances integrin-mediated adhesion and invasion by stimulating c-src (a raft resident) activity, and this effect is reversed when the lipid rafts are disrupted [59]. In addition, CD24 can recruit phosphorylated Met into lipid rafts to increase ATP-binding cassette (ABC) transporters expression, resulting in cisplatin resistance [60].

CD44 is a cell surface hyaluronan receptor with signaling functions [61, 62] that maintains the stemness of CSCs [63]. Moreover, CD44 is also highly expressed in various CSCs and is a common CSC marker in solid tumors such as pancreatic and prostate cancers [31, 64, 65]. Previous studies revealed that CD44 exists in lipid rafts [66–68]. Concretely, lipid rafts are isolated using detergent extraction methods combined with isopycnic sucrose density gradient fractionation. Western blotting assay and co-immunoprecipitation detection manifest the expression of CD44 is dramatically higher in lipid raft fractions than in non-lipid raft fractions [68]. Likewise, immunofluorescence observation further shows that CD44 co-localizes with the raft marker caveolin-1 [69]. Meanwhile, the functions of CD44 are dependent on the CD44 cluster to lipid rafts [70]. Following the interaction of hyaluronan with its receptor, CD44 multiprotein complexes assemble in lipid rafts to activate downstream signaling, which is critical for the properties of CSCs [17, 71]. In contrast, lipid raft disruption by depleting cholesterol prevents CD44 from recruiting to lipid rafts, enhances CD44 shedding, and suppresses CD44-dependent cancer cell migration [72].

CD133 (alias prominin-1/AC133), a pentaspan transmembrane glycoprotein, is a marker for stem cells and CSCs, which rarely appears on normal tissue cells [36, 37, 51]. In normal stem cells, CD133 is mainly associated with cell proliferation [73], while in CSCs, it is involved in cancer cell proliferation, carcinogenesis, metastasis, recurrence, and chemoresistance [74, 75]. This critical CSC marker is localized in lipid rafts [14, 76]. CD133 induces EMT and stemness properties by interacting with the Src (lipid raft-related protein) [77]. Similarly, CD133 can activate raft resident Src to phosphorylate focal adhesion kinase (FAK), promoting cell migration [78]. Furthermore, the formation of polarized CD133/ integrin induces lipid raft coalescence and then activates Src to promote β-catenin nuclear translocation and CSC self-renewal [79]. Notably, perturbed lipid raft coalescence abolishes β-catenin activation and the CSC phenotype.

CXCR4 is a G-protein-coupled chemokine receptor for stromal cell-derived factor 1 (SDF-1, also known as CXCL12) and is a crucial CSC marker [80–83]. Increased expression of CXCR4 correlates with chemotaxis, invasiveness, and CSC properties. Downregulated CXCR4 inhibits cancer cell stemness [84]. Studies found that CXCR4 and c-MET (MET proto-oncogene) colocalize with caveolin-1 in lipid rafts, and caveolin 1 is required for CXCL12/ CXCR4-induced c-MET activation that enhances EMT [85]. Ultimately, disruption of lipid rafts blocks CXCL12/ CXCR4 activation [85]. That is, CXCR4 cannot work without lipid rafts.

In sum, CSC markers are present in lipid rafts, and their function is regulated by lipid rafts. The therapeutic antibodies targeting CSC surface markers have been approved for clinical application. Hence, lipid rafts may assist these molecular targeted therapies. Targeting lipid rafts may lead to dysfunction of CSC markers, which also offers exciting possibilities for clear CSCs.

## CSC self-renewal and lipid raft

Self-renewal is the ability of stem cells that divide to produce more stem cells [86]. That is why these distinct subpopulations of cancer cells are defined as CSCs. The self-renewal capacity of CSCs is aggressive and uncontrollable, while that of the normal stem cells is orderly. CSC self-renewal involves a lot of signaling pathways, primarily including the Wnt/ β-catenin, Notch, and Hedgehog pathways [87–89]. As a platform for cell signaling transduction, lipid rafts control CSC self-renewal signaling pathways.

The Wnt/ β-catenin pathway is one of the significant signaling pathways involved in stem cell self-renewal. The positive effect of the Wnt/β-catenin pathway on CSC self-renewal has been reported in various cancers [90, 91], such as liver cancer [90], breast cancer [92], colorectal cancer [93], and acute myelogenous leukemia [94, 95]. Wnt ligands can combine with their receptors Frizzled receptors (Fz) and co-receptors low-density lipoprotein receptor-related protein 5 (LRP5)/6, phosphorylating the cytoplasmic domain of LRP and then recruiting the scaffolding protein Disheveled (Dvl) to disassemble the destruction complex. The destruction complex mediates the β-catenin degradation, which includs scaffolding proteins Axin, adenomatous polyposis coli (APC), the kinase proteins glycogen synthase kinase-3β (GSK-3β), and casein kinase 1α (Ck1α). As a result, β-catenin accumulates and activates numerous target gene transcription [96, 97]. However, the binding of Wnt ligand to their receptors Fz8 and LRP 6 predominantly occurs in ordered lipid raft microdomains because Wnt receptor complexes are partially located in lipid rafts [98–100]. The more receptors in lipid rafts, the higher the activity of Wnt/ β-catenin signaling (Data from simulation experiments [101]). In addition, removing LRP6 from lipid rafts to the non-lipid rafts domain suppresses the β-catenin pathway [99], while increasing lipid raft mobility can enhance Wnt-mediated signaling [102]. Collectively, these imply that lipid rafts can regulate CSC self-renewal by mediating the Wnt/β-catenin pathway.

Another way to regulate CSC self-renewal is Hedgehog (Hh) pathway [103]. The Hh pathway can be triggered by Sonic Hedgehog (SHH), Indian Hedgehog (IHH), and Desert Hedgehog (DHH). Upon binding of the above ligands to their receptor Patched (Ptc), and Ptc released the repression on Smoothened (Smo) to allow GLI (a full-length form of glioma-associated oncogene homolog) to enter the nucleus that stimulates downstream target genes transcription (such as PTCH1, GLI1, and Wnt) [104, 105]. Activation of the Hh pathway facilitates CSC self-renewal [106]. However, lipid rafts are critical for high Hh activity transduction because the receptors Ptc and Smo are located in lipid rafts upon Hh stimulation [107–109]. Furthermore, when lipid rafts are disrupted, Hh pathway activity is downregulated, and the higher-order clusters of Smo on the plasma membrane are significantly reduced [107]. Another study has shown that lipid rafts are related to the intracellular transportation of Shh. Specifically, intracellular Shh can form protein complexes with caveolin-1 in the Golgi apparatus to transport lipid raft microdomains [110]. These suggest lipid rafts involve signal transduction and intracellular ligand transportation of Hh signaling, affecting CSC self-renewal.

Further, the Notch pathway is also essential for stem cell self-renewal. There are five Notch ligands (Delta-like (DLL) 1, DLL3, DLL 4, Jagged (JAG) 1, JAG 2) and four receptors (Notch1-Notch4) in mammals. Upon their interaction, proteolytic events of disintegrin and metalloproteinases (ADAMs)-mediated cleavage at the S2 site and γ-secretase-mediated cleavage at the S3 site of the Notch receptor release the Notch intracellular domain (NICD) and enters the nucleus to activate downstream Notch target gene expression [111, 112], governing CSC self-renewal and differentiation [113, 114]. Interestingly, lipid rafts are involved in the action of the protease γ-secretase. The protease γ-secretase assembles into lipid rafts and exhibits the highest activity there, indicating γ-secretase function depends upon its distribution between the raft and non-raft microdomains [115, 116]. Further studies have shown that hypoxia-induced “raft clusters” promote Notch3 partially migrated into lipid rafts, where they interact with γ-secretase, leading to the progression of human prostate cancer by stimulating Notch signaling [117]. Moreover, lipid rafts resident caveolin-1 is related to Notch-1expression. Downregulation of caveolin-1 decreases Notch-1 and NICD expression and inhibits Notch signaling that promotes stem cell differentiation [118]. Meanwhile, Ethanol may inhibit γ-secretase proteolytic activity that suppresses Notch signaling, possibly by decreasing caveolin-1 [119]. Overall, lipid rafts and their associated protein caveolin-1 regulate the downstream cascade of the Notch pathway that affects CSC self-renewal.

Apart from the above-mentioned pathways for CSC self-renewal need lipid rafts. The essential CSC self-renewal protein CD55, an intrinsic cell surface GPI-anchored membrane complement inhibitor that protects cells from complement-mediated lysis [120], also requires GPI-anchoring to lipid rafts for CSC stemness maintenance and cisplatin resistance [18].

CSC self-renewal is critical for cancer propagation. As discussed above, lipid rafts mediate CSC self-renewal, which strongly supports that understanding and manipulating lipid rafts in the plasma membrane holds promise for simultaneously inhibiting three CSC self-renewal related pathways. Our previous study showed that inhibiting lipid raft structural protein caveolin-1 could repress CSC properties by blocking the Wnt/ β-catenin signaling pathway [15], which proves the feasibility of the above perspective.

## Drug resistance and lipid raft

Quiescent CSCs are a recognized cause of therapeutic resistance [121]. Cell surface signals control stem cell hibernation or cell cycle re-entry [122]. Lipid rafts are vital for stem cell fate as the signal hub. For example, lipid raft clustering regulates the fate of hematopoietic stem cells (HSC) in vitro [123]. Therefore, eliminating quiescent CSCs is one approach to preventing drug resistance, such as the selective eradication of quiescent human leukemia stem cells by targeting BCL 2-dependent oxidative phosphorylation [124]. In contrast, keeping CSCs in a permanent quiescent state is another approach. As long as CSCs do not enter the cell cycle, their presence does not affect cancer progression. Excitingly, diphenyleneiodonium chloride can induce CSCs quiescence phenotype to make CSC hibernation [125], and this study brings hope for CSCs quiescence strategies. Intriguingly, the inhibition of lipid raft aggregation induces cyclin-dependent kinase inhibitor p57 (Kip2) expression, leading to HSCs hibernation [126], which is theoretically effective to CSCs, based on their similarity to stem cells. That means that we can prohibit lipid raft clusters to keeping CSCs quiescent. Among potential candidates, emodin has been reported to suppress lipid raft clusters [127]. The future will reveal whether such a bold idea will be implemented.

Drug efflux pumps (e.g., ABC transporter) are another mechanism driving drug resistance [128, 129], which expel anti-cancer drugs from CSCs. For example, CD133^+^ melanoma CSCs resist caffeic acid phenethyl ester because of elevated ABCB5 expression [130]. Likewise, ABCB5 increases resistance in CD133^+^ glioblastoma multiforme CSCs, and ABCB5 inhibition makes CSCs sensitive to temozolomide [131]. Importantly, these multidrug resistance-associated drug efflux transporters are found in lipid rafts [132], implying that lipid rafts can regulate these resistance-related proteins. Numerous studies confirmed that disrupting lipid rafts can reverse drug resistance. For example, interference with lipid rafts abrogates ABC transporters induced chemoresistance in CD133^+^ pancreatic CSCs [14]. Depletion of lipid rafts with simvastatin suppresses integrin-β3/FAK signaling, re-sensitizing cancer cells to paclitaxel [133]. Similarly, disrupting the key lipid raft proteins, flotillins, reverses drug resistance in colorectal cancer cells [134].

## EMT and lipid raft

EMT is a cell-biological program in which the epithelial phenotype is lost and mesenchymal characteristics are acquired [135]. EMT is generally accepted to be a critical step in the migration and invasion of cancer cells [136]. In the context of CSC theory, EMT programs have been demonstrated to promote CSC stemness [137, 138] or rather generate CSCs [139]. For example, most mesenchymal-like human mammary epithelial cells become CD44^+^/CD24^−^ neoplastic mammary stem cells after undergoing EMT [139]. Lipid rafts are indispensable for the whole EMT process. EMT-induced motility and stem cell properties require the destabilization of lipid rafts. Generally, lipid raft stability is quantified by measuring miscibility transition temperature: 50% of the vesicles are phase separated. In concrete terms, cells that undergo EMT have reduced phase separation in isolated plasma membrane vesicles, which are divided into liquid-ordered (raft) domain and disordered (non-raft) domain, indicating that cells in the epithelial state possess more stable lipid raft domains. Cell migration, mammosphere formation, and alternate splicing from a variant isoform (CD44v) in epithelial cells to a standard isoform (CD44s) in mesenchymal cells are inhibited when DHA is treated to stabilize raft phase separation, suggesting that EMT and stemness are suppressed [140].

Transforming growth factor-β (TGF-β) is a potent inducer that drives EMT mainly through the canonical Smad-dependent signaling pathway [141]. TGF-β combines with the TGF-β receptor II (TGF-βRII) to phosphorylate TGF- β receptor I(TGF-βRI). Subsequently, R-smad (receptor-regulated Smad) are activated to form R-Smad/ CO-Smad (common partner Smad) complexes and translocate to the nucleus, where they regulate the transcription of EMT target genes. I-Smad (inhibitory Smad) can interact with TβRI to inhibit TGF-β signaling through ubiquitin-dependent degradation [141, 142]. A previous study has found that TGF-βRII is partially localized in lipid rafts and is internalized for degradation in an I-smad-dependent manner. In particular, TβRII and caveolin-1 colocalize in Mv1Lu cells expressing extracellularly HA-tagged TGF-β type II receptors by three-color immunofluorescence analysis. Similarly, immunoprecipitation and western blotting analysis have also yielded similar results. Furthermore, caveolin-1 can cooperate with Smad7–Smurf2 to enhance receptor degradation in cells transiently transfected with TβRI, TβRII, and Smad7, as detected by immunoprecipitation and immunoblotting assay. The t_1/2_ of TGF-β receptor is 6 h in cells without caveolin-1 expression, while the t_1/2_ is reduced to 4 h in cells expressing caveolin-1 [143]. Meanwhile, this I-smad-dependent receptor turnover is blocked when lipid rafts are disrupted [143]. Likewise, the anti-cancer drug sorafenib suppresses TGF-β signaling by inducing caveolae/lipid raft-mediated TGF-βRII degradation, thereby inhibiting EMT [144]. The evidence above indicates lipid rafts negatively regulate TGF- β signaling by promoting TGF-βRII degradation. Conversely, there is a clue that lipid rafts facilitate TGF-β signaling, as lipid rafts seem to provide a platform for TGF-βRII to activate TGF-βRI. TGF-βRI and TGF-βRII are imaged and tracked in living cells by single-molecule imaging. The mobility of TGF-βRI is obviously declined after TGF-β stimulation, which is attributed to the formation of TGF-βRI/ TGF-βRII/ TGF-β signaling complex. However, in cells whose lipid rafts are disrupted by nystatin or MβCD, the diffusion rate of TGF-βRI is not altered by TGF-β treatment, and the phosphorylation level of downstream Smad2 is decreased [145]. In brief, lipid rafts play a role in TGF- β/ Smad signal, yet whether lipid rafts play a positive or negative role during this process requires further investigation. In addition, lipid rafts also positively affect non-canonical TGF- β pathway. Activation of the non-canonical TGF-β/ MAPK (mitogen-activated protein kinase) pathway is dependent on lipid rafts [146]. The disruption of lipid rafts disturbs the localization of TGF-β receptors in lipid rafts and impairs TGF-β–mediated MAPK activation, thereby inhibiting EMT [146].

The lipid raft marker flotillin is also involved in the EMT process [8]. After radiofrequency ablation therapy, the residual hepatocellular carcinoma cells progress rapidly, probably because the residual cell acquires stemness through EMT. Studies have attributed this phenomenon to the upregulation of flotillin-1 and flotillin-2, which promote EMT by activating the Akt/ Wnt/ β-catenin signaling pathway [147]. In early-stage cervical cancer, flotillin-1 is highly expressed and motivates EMT through Wnt/ β-catenin and NF-κB signaling pathway [148]. Likewise, flotillin-2 is upregulated in nasopharyngeal carcinoma and enhances EMT by activating lipid raft resident protein Src, which might be served as a downstream factor of TGF- β, indicating that lipid rafts are closely involved in this process [149].

Notably, the EMT inducer TGF-β has efficiently promoted non-CSC-to-CSC conversion [150]. That is, TGF-β induces CSC plasticity. These interconversions between epithelial non-CSC and mesenchymal/ CSC states proved the impact of EMT on CSC. Lipid raft and its marker flotillin facilitate EMT. That indicates we can prevent non-CSCs poised to become CSCs by disrupting lipid rafts.

## CSC niche and lipid raft

The stem cell niche is a dynamic microenvironment surrounding stem cells and is made up of adjacent cells, cytokines, and the extracellular matrix [155]. It is a subcompartment within the tumor microenvironment (TME). Like normal stem cells, CSCs live in niches [156]. CSCs are tightly dependent on their niche, which provides a favorable living environment for CSCs. In particular, lipid rafts transmit signals for the cells in the CSC niche to maintain CSC niche function.

CSCs promote niche angiogenesis and enhance self-maintenance by secreting vascular endothelial growth factor A (VEGF) [157, 158]. VEGF stimulates angiogenic ERK (extracellular regulated protein kinases)/MAPK signaling by binding to VEGF receptor-2 (VEGFR-2). VEGFR-2 has been reported to be located in lipid rafts, and lipid raft disruption can selectively reduce VEGFR2 levels and inhibit the downstream ERK/ MAPK pathway [151].

The ECM, including fibrous proteins, glycoproteins, proteoglycans, and polysaccharides, is an essential structural component of the CSC niche [159]. It can physically shield CSCs from therapeutic agents and modulate CSCs [160]. Integrins are prominent ECM surface receptor proteins that mediate cell-to-cell interactions and communication with the ECM [161], which are critical for the function of CSC [162, 163]. The role of integrin depends a lot on lipid raft to transmit signals. For example, lipid rafts regulate the initial spread of cancer cells by recruiting and modifying adaptor proteins (such as talin, α-actinin, vinculin, paxillin, and FAK) to binding β1 integrins [164]. Notably, lipid raft structure disruption prevents β1 integrin clustering [152].

CAFs are stromal cells of the CSCs niche that sustain cancer stemness and regulate CSCs plasticity via paracrine signaling [165–168]. SDF-1/CXCL12 is the primary secretome of CAFs [169], which promotes cancer growth by interacting with CXCR4 in cancer cells [170, 171]. The function of CXCL12 is recognized to be mediated by lipid rafts [172]. CXCL12 is recruited to lipid rafts, and its operation requires the involvement of raft resident Src-family protein tyrosine kinases [172]. Furthermore, inhibition of lipid rafts blocks CXCL12 signaling [153, 173].

Cellular senescence is the latest addition to the hallmarks of cancer [174]. Treatment-induced senescent cancer cells or other senescent cells in the TME can remodel microenvironments to promote cancer phenotypes through proinflammatory senescence-associated secretory phenotype (SASP) in a paracrine manner [175–178]. Meanwhile, SASP facilitates CSCs by providing chemokines and inflammatory cytokines (such as IL-1β, IL-8, and CXCR1) [179, 180]. The recently published review by our group revealed that lipid rafts and their component protein are integral parts of cellular senescence [154]. For example, caveolin-1 induces senescence by activating P53 though affecting Mdm2 (mouse double minute 2 homolog), PP2A-C (protein phosphatase 2A-C subunit), Sirt1, TrxR1 (thioredoxin reductase 1), Nrf2 (nuclear factor erythroid-2-related factor-2), and EGFR (epidermal growth factor receptor) [154]. It is feasible to manipulate lipid rafts to suppress cellular senescence and minimize the effects of SASP on the CSC niche.

As long as the niche remains intact, CSCs continue to be recreated to maintain cancer homeostasis [181]. The efficient functioning of the CSC niche requires the collaborate of different cells, and cell communication is the way of cooperation. As the signal hub of cells that receive and transmit signals, the absence of lipid rafts would break the niche balance. Considering lipid raft regulates support signals of CSC niche, it might be the one to terminate CSCs.

## Lipid raft disrupted agents and anti-CSC strategies

**Fig. 2:**
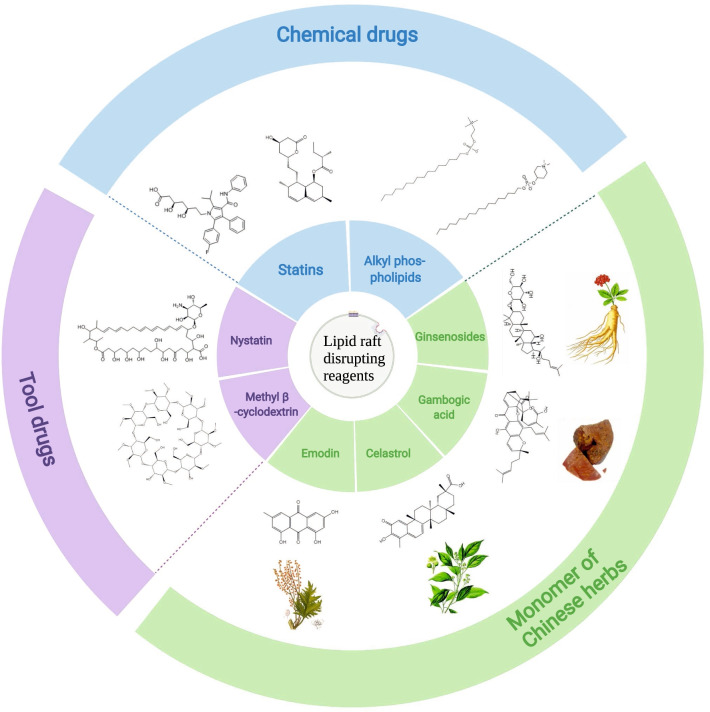
Lipid raft disrupting reagents. Methyl β -cyclodextrin and nystatin are tool drugs for the disruption of lipid rafts. Statins and alkyl phospholipids are chemicals that can interrupt the integrity of lipid rafts. Moreover, emodin, celastrol, ginsenosides, and gambogic acid are all extracted from the herb

In current therapeutic strategies to eradicate cancer, the combination of traditional anti-cancer drugs and CSC-targeted agents simultaneously kills both CSCs and non-CSCs, preventing cancer recurrence. Targeted lipid raft can thus be a sally port for CSC elimination. That is, the combination of lipid raft-targeted drugs and anti-cancer drugs could eradicate cancer. Therefore, agents targeting lipid rafts (also known as raftophilic) show great anti-cancer potential (Fig. 2). In addition to the tool drugs MβCD and cholesterol-sequestering agent nystatin, the major types of lipid raft disrupting agents are as follows.

Cholesterol synthesis inhibitor statins disrupt lipid raft by depleting cellular cholesterol. Studies have manifested that lovastatin, one of the statins, decreases the metastatic potential of pancreatic CSCs and increases chemosensitivity by disrupting lipid rafts [14]. Likewise, gambogic acid impairs lipid rafts and inhibits cancer cell adhesion by reducing cellular cholesterol content [182]. Moreover, the cholesterol analogs ginsenosides disrupt lipid rafts by increasing membrane fluidity [183]. The ginsenoside derivative Rp1 has been reported to reverse multidrug resistance by modulating lipid rafts to inactivate ABCB1 (drug effluxes protein in lipid raft) and Src [184]. Endogenous phospholipids analogs, alkyl phospholipids (ALPs), and modified APL derivatives include edelfosine, miltefosine, perifosine, and erufosine interrupt lipid raft integrity by incorporating into lipid raft, thereby inducing cancer cell growth arrest and apoptosis [4, 185–192]. Miltefosine preferentially induces colorectal CSCs death by disrupting lipid raft [13], and the lipid raft-targeted edelfosine has been recently reported to induce apoptosis in pancreatic CSCs by autophagy inhibition [193]. In addition, the major active component of the rhizome of Rheum palmatum L, emodin, has been found to inhibit the lipid raft clustering by reducing cholesterol and sphingolipids, inhibiting cancer cell adhesion [127]. Our group initially demonstrated that celastrol, a pentacyclic triterpene extracted from Tripterygium wilfordii Hook F, decreases caveolae (the subset of lipid raft), resulting in declined stemness of CD133^+^ CSCs [15]. Targeting lipid raft therapy could eliminate CSCs, eventually resolving many pending issues.

## Conclusion and perspective

A complete cure for cancer is the common pursuit of humanity, while CSC stands in the way. Existing evidence demonstrates lipid rafts are associated with CSC self-renewal, quiescence, EMT, and CSC niche. As a platform for protein anchorage, more than half of CSC markers are located in lipid rafts, and drug efflux proteins that contribute to CSC resistance are also present in lipid rafts. That means lipid rafts may regulate these raft-relevant proteins. In particular, lipid rafts regulate CSC self-renewal and EMT by mediating Wnt/ β-catenin pathway, Notch pathway, Hh pathway, and TGF-β pathway. Furthermore, lipid rafts have the potential to intervene in CSC quiescence, and they are also responsible for cellular communications of the CSC niche. Therefore, lipid raft might be an effective target for CSC elimination (Table 2).

**Table 2:** Role of lipid raft

	Proteins located in lipid raft	Cell lines	The effect of lipid raft	Lipid raft disrupting reagents	References
CSC markers	CD24	MTLY	Promotes FAK/integrin-mediated adhesion and invasion	MβCD (methyl-β-cyclodextrin)	[59]
		CD24^+^ hec-1A and hec-108	Promotes drug resistance	N/A	[60]
	CD44	U-251 MG	Promotes cancer cell migration	MβCD, simvastatin	[72]
	CD133	CD133^+^ SAS and OECM1	Promotes EMT and maintains CSC properties	PP2 (inhibitor of Src activity)	[77]
		CD133^+^ U87MG	Promotes CSC self-renewal	MβCD, knockdown of Par3 and Par6 (perturbed lipid raft coalescence and cell polarization)	[79]
	CXCR4	MGC-803	Promotes EMT	Nystatin, knockdown of caveolin-1	[85]
CSC self-renewal	Fz receptor, LRP	HEK293T	Promote Wnt/ β-catenin pathway	Cholesterol oxidase (deplete cholesterol), myriocin (deplete sphingomyelin), oseltamivir (deplete GM1 ganglioside), membrane cholesterol‐deficient Niemann–Pick C disease cells	[98]
	Ptc, Smo,	*Drosophila* S2 cell	Promotes Hedgehog pathway	Eicosapentaenoic acid 20:5(n-3), PUFA (disrupt lipid raft stability)	[107]
	Ptc, Caveolin-1	Chinese hamster ovary cells	Promotes Hedgehog pathway	MβCD	[108]
	γ-secretase, Notch3	LNCaP	Promotes Notch pathway to increase cancer progression	N/A	[117]
	Caveolin-1	Wistar rats MSCs	Promotes Notch pathway by maintaining Notch-1expression	N/A	[118]
Drug resistance	Lipid raft	A549T	Promotes EMT-associated drug resistance	Simvastatin	[14]
	Flotillins	HCT-15	Promotes drug resistance	MβCD, knockdown of flotillin-1 and flotillin-2	[134]
EMT	TGF-βRII	Mv1Lu, R1B, HepG2	Mediated TβR-II degradation to inhibit EMT by TGF/Smad pathway	Cholesterol, MβCD, nystatin	[144]
	TGF-βRII and TGF-βRI	HeLa	Promotes TGF-βRII activate TGF-βRI to facilitate EMT by TGF/Smad pathway	MβCD, nystatin	[145]
	Flotillins	HCCLM3	Promotes EMT and metastasis by activating the Akt/ Wnt/ β-catenin pathway	Knockdown of flotillin-1 and flotillin-2	[147]
	Flotillin-1	Primary Hkc, HeLa, C33A, SiHa	Promotes metastasis through Wnt/ β-catenin and NF-κB pathway-regulated EMT	Knockdown of flotillin-1	[148]
	Flotillin-2, Src	CNE-1, 6–10B	Promotes metastasis through TGFβ-induced EMT	Knockdown of flotillin-2	[149]
CSC niche	VEGFR-2	BAECs	Promote angiogenesis by ERK/ MAPK pathway	MβCD, sphingomyelinase, simvastatin	[151]
	β1 integrin	A375	Facilitating β1 integrin clustering to promote focal adhesion formation	MβCD	[152]
	CXCL4, Rac1	EC, CE 48 T/VGH, CE 81 T/VGH, CE 146 T/VGH	Promote SDF-1α-induced invasion by Rac1/ PI3K/ Akt pathway	N/A	[153]
	Caveolin-1	NIH 3T3 cells, MEFs, etc.	Promote cellular senescent to build a CSC supporting microenvironment	Knockout of caveolin-1	[154]

However, there are still numerous challenges in targeting the lipid raft in CSCs. How to achieve accurate drug delivery of lipid rafts in CSCs to minimize side effects remains to be determined. Although caveolin-1 and flotillin are generally recognized markers of lipid rafts [8], there are no specific markers of lipid rafts for CSCs. CSCs can be distinguished from stem cells or cancer cells if these lipid raft markers are found. An example that matches this point is the differential expression of raft-associated proteins (namely caveolin-1, flotillin-1, vimentin, galectin-3, and glyceraldehyde-3-phosphate dehydrogenase) can clearly distinguish stem cells and leukemia cells [194]. Exploring lipid raft-specific markers in CSCs that differentiate from other cells, such as cancer cells or normal stem cells, would facilitate targeted and accurate drug delivery of lipid rafts. For example, it contributes to the preparation of chemical immune conjugates, which are monoclonal antibodies that recognize CSC lipid raft-specific markers coupled with lipid raft disrupting agents.

The lipid rafts content of CSCs is higher than that of cancer cells [13]. What it means for CSCs is unclear, but it is apparent that lipid rafts contain functional CSC markers and signaling proteins necessary for CSCs. Is this elevated lipid raft phenomenon related to CSC hyperfunction? It is still a mystery. However, there is no doubt that lipid rafts are beneficial to CSCs. In turn, lipid raft disruption is detrimental to CSCs. CSCs can be cleared once targeting lipid rafts on CSCs is resolved. Interestingly, the miltefosine mentioned above preferentially targets higher lipid raft cells [13]. Lipid rafts are abundant on the membranes of CSCs, which is valuable in their elimination. Unfortunately, normal stem cells also have high lipid raft content [195]. To date, few studies compare the lipid raft content of CSCs and normal stem cells. If CSCs have a higher lipid raft content than normal stem cells, it is hypothesized that ALPs drugs can be utilized to target CSCs directly. But if CSCs have a lower or similar lipid raft content than normal stem cells, CSCs must be distinguished from normal stem cells when using ALPs. Therefore, once the selectivity issue between CSCs and normal stem cells is overcome, targeting the CSC lipid raft is feasible. Recent research has already demonstrated the ability to isolate lipid rafts from CSCs [16], which provides a technical guarantee for further investigation. The scent of victory is in the air.

## Data Availability

Not applicable.

## Abbreviations

ABC: ATP-binding cassette
ADAMs: A disintegrin and metalloproteinases
ALDH: Aldehyde dehydrogenase
ALPs: Alkyl phospholipids
BCL2: B-cell lymphoma-2
CAFs: Cancer-associated fibroblasts
c-MET: MET proto-oncogene
CSCs: Cancer stem cells
CtxB: Conjugated cholera toxin B
CXCR4: Chemokine receptor-4
DHH: Desert Hedgehog
DLL: Delta-like
ECM: Extracellular matrix
EGFR: Epidermal growth factor receptor
EMT: Epithelial-mesenchymal transition
EpCAM: Epithelial cell adhesion molecule
ERK: Extracellular regulated protein kinases
FAK: Focal adhesion kinase
Fz: Frizzled receptors
GLI: Glioma-associated oncogene homolog
GPI: Glycosylphosphatidylinositol
Hh: Hedgehog
HSC: Hematopoietic stem cells
IHH: Indian Hedgehog
JAG: Jagged
LRP: Lipoprotein receptor-related protein
MAPK: Mitogen-activated protein kinase
Mdm2: Mouse double minute 2 homolog
NICD: Notch intracellular domain
Nrf2: Nuclear factor erythroid-2-related factor-2
PP2A-C: Protein phosphatase 2A-C subunit
Ptc: Patched
SASP: Senescence-associated secretory phenotype
SDF-1/ CXCL12: Stromal cell-derived factor 1
SHH: Sonic Hedgehog
Smo: Smoothened
TGF-β: Transforming growth factor-β
TGF-βRI: TGF-β receptor I
TGF-βRII: TGF-β receptor II
TME: Tumor microenvironment
TrxR1: Thioredoxin reductase 1
VEGF: Vascular endothelial growth factor A
VEGFR-2: VEGF receptor-2
